# Hybrid Poly(*β*‐amino ester) Triblock Copolymers Utilizing a RAFT Polymerization Grafting‐From Methodology

**DOI:** 10.1002/macp.202300262

**Published:** 2023-11-07

**Authors:** Karolina Kasza, Amr Elsherbeny, Cara Moloney, Kim R. Hardie, Miguel Cámara, Cameron Alexander, Pratik Gurnani

**Affiliations:** ^1^ Division of Molecular Therapeutics and Formulation School of Pharmacy University of Nottingham Nottingham NG7 2RD UK; ^2^ National Biofilms Innovation Centre School of Life Sciences, Biodiscovery Institute University Park, University of Nottingham Nottingham NG7 2RD UK; ^3^ Ex Vivo Cancer Pharmacology Centre of Excellence School of Medicine University of Nottingham Nottingham NG7 2RD UK; ^4^ School of Medicine Biodiscovery Institute University Park, University of Nottingham Nottingham NG7 2RD UK; ^5^ UCL School of Pharmacy University College London 29–39 Brunswick Square London WC1N 1AX UK

**Keywords:** biomaterials, grafting‐from, hydrophobic drug delivery, poly(*β*‐amino esters), RAFT, Triblock copolymer

## Abstract

The biocompatibility, biodegradability, and responsiveness of poly(*β*‐amino esters) (PBAEs) has led to their widespread use as biomaterials for drug and gene delivery. Nonetheless, the step‐growth polymerization mechanism that yields PBAEs limits the scope for their structural optimization toward specific applications because of limited monomer choice and end‐group modifications. Moreover, to date the post‐synthetic functionalization of PBAEs has relied on grafting‐to approaches, challenged by the need for efficient polymer–polymer coupling and potentially difficult post‐conjugation purification. Here a novel grafting‐from approach to grow reversible addition–fragmentation chain transfer (RAFT) polymers from a PBAE scaffold is described. This is achieved through PBAE conversion into a macromolecular chain transfer agent through a multistep capping procedure, followed by RAFT polymerization with a range of monomers to produce PBAE–RAFT hybrid triblock copolymers. Following successful synthesis, the potential biological applications of these ABA triblock copolymers are illustrated through assembly into polymeric micelles and encapsulation of a model hydrophobic drug, followed by successful nanoparticle (NP) uptake in breast cancer cells. The findings demonstrate this novel synthetic methodology can expand the scope of PBAEs as biomaterials.

## Graphical abstract

To address the limited scope for poly(*β*‐amino ester) (PBAE) structural optimization a novel synthetic grafting‐from methodology is developed to grow reversible addition–fragmentation chain transfer (RAFT) polymers from a PBAE scaffold. The potential biological applications of the resulting ABA block copolymers are then illustrated through assembly into polymeric micelles and encapsulation of a model hydrophobic drug.

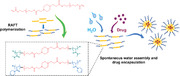

## 1 Introduction

Poly(*β*‐amino esters) (PBAEs) have recently attracted considerable attention due to their inherent biocompatibility, biodegradability, responsiveness, and structural versatility.^[^ ^1^ ^]^ Synthesized through a one‐pot aza‐Michael addition of primary or secondary amines with diacrylates, PBAEs are equipped with hydrolytically degradable ester bonds supporting their use as biomaterials. Furthermore, the inherent tertiary amine groups permit their use for carrying negatively charged cargo or as pH responsive materials.^[^ ^2^ ^]^ Accordingly, these advantages mean PBAEs have been widely explored in areas such as drug, gene, and vaccine delivery, particularly when assembled into micelles or polyelectrolyte complexes.^[^ ^1^, ^3^, ^4^, ^5^, ^6^, ^7^, ^8^ ^]^

However, the step‐growth polymerization mechanism that yields PBAEs means that structure optimization toward specific applications must either come from the limited amine and diacrylate monomer choice or via end‐group modification. Meanwhile other polymerization techniques, such as controlled radical polymerizations or ring opening polymerizations, can easily control block length and sequence for nanoparticle formulation. This is particularly significant for tuning the surface chemistries of nanomaterials in applications where stabilization in physiological fluids or targeting to specific cell types is required.^[^ ^8^, ^9^ ^]^ Surprisingly, no simple strategy to tune the surface chemistries for PBAE nanoparticles has yet been reported, despite their widespread use as a biomaterial. The current state of the art relies on post‐polymerization grafting of pre‐synthesized polymers such as poly(ethylene glycol) (PEG)^[^ ^10^, ^11^ ^]^ onto reactive handles at the chain end of prepared PBAEs, with copolymerization shown to enhance stability and promote efficacy in both the context of gene and drug delivery. Kim et al.^[^ ^10^ ^]^ reported the synthesis of PEG–PBAE–PEG triblock copolymers, for gene delivery, with a prolonged particle stability and improved efficacy observed in vivo. Thambi et al.^[^ ^12^ ^]^ demonstrated a methodology to obtain PBAEs with hyaluronic acid attached, with an improvement in doxorubicin efficacy and a decrease in toxicity reported. Moreover, Cordeiro et al.^[^ ^13^ ^]^ reported high‐buffering capacities and efficient pDNA delivery for (2‐(dimethylamino)ethyl methacrylate)‐PBAE‐(2‐(dimethylamino)ethyl methacrylate) triblock copolymers. In each case, polymer attachment was undertaken through a grafting‐to approach, relying on efficient polymer–polymer coupling and potentially challenging post‐conjugation purification. We postulated that a grafting‐from approach to grow polymers from a PBAE scaffold would enable access to a new class of PBAE, expanding their scope in biomedical applications.

Grafting‐from strategies have previously been explored with compounds such as cyclic peptides,^[^ ^14^ ^]^ cellulose,^[^ ^15^ ^]^ and proteins^[^ ^16^ ^]^ primarily utilizing reversible deactivation radical polymerization techniques. Of these, reversible addition–fragmentation chain transfer (RAFT) polymerization has been especially popular for grafting‐from methodologies due to its molar mass control, ambient polymerization conditions, monomer versatility, orthogonality with common functional moieties, and importantly, ability to create macroinitiators to extend.^[^ ^17^ ^]^

Therefore, in this study we propose a new class of block copolymer produced via a grafting‐from methodology utilizing RAFT polymerization to functionalize PBAEs. Hence, we enable synthesis of PBAE triblock copolymers, utilising a PBAE core with a versatile range of graft polymers both ends of the PBAE chain, therefore expanding the scope of PBAEs as biomaterials. Initially, we describe the development of PBAE macromolecular chain transfer agents through a multi‐step capping procedure, followed by RAFT polymerization with a range of monomers to produce PBAE–RAFT hybrid triblock copolymers. Following successful synthesis, we then illustrate their potential biological applications through assembly into polymeric micelles, encapsulation of a model hydrophobic drug and demonstration of successful nanoparticle (NP) uptake in breast cancer cells.

## 2 Experimental Section

### 2.1 Materials

Butanediol diacrylate (BDD), hexanediol diacrylate (HDD), 3‐aminopropanol (3AP), piperazine (PIP), triethylamine (TEA), 2‐acrylamido‐2‐methylpropane sulfonic acid (AMPS), dimethyl sulfoxide (DMSO)‐d6 (99.5% D atom), chloroform‐d (99.8% D atom), 4,4′‐azobis(4‐cyanovaleric acid) (ACVA, >98%), docetaxel (DTX) (99.8% pure), Roswell Park Memorial Institute media (RPMI), trypsin‐EDTA solution, fetal bovine serum (FBS), l‐glutamine were obtained from Sigma‐Aldrich without further purification. *N*‐Acryloyl morpholine (NAM), *N*,*N*‐dimethylacrylamide (DMA), acrylic acid (AA), and 2‐(*N*,*N*‐dimethyl amino) ethyl acrylate (DMAEA) were all purchased from Sigma‐Aldrich and the inhibitor removed by passing the monomers through a column of basic aluminum oxide. Formvar carbon 200 mesh and graphene oxide grids were purchased from Agar Scientific. PrestoBlue Cell Viability Reagent was purchased from ThermoFischer Scientific. LumiTracker Lyso Green fluorescent dye was purchased from Lumiprobe. Acryloxyethyl thiocarbamoyl Rhodamine B was purchased from Polysciences, Inc. Solvents and other reagents were acquired from commercial sources and used as received unless stated otherwise.

2‐(((Butylthio)carbonothioyl)thio)propanoic acid (PABTC) and *N*‐hydroxysuccinamide‐(propanoic acid)yl butyl trithiocarbonate (NHS‐PABTC) were synthesized by methods previously reported in literature.^[^ ^14^, ^18^ ^]^

### 2.2 Methods

#### 2.2.1 Instrumentation and Analysis

##### NMR Spectroscopy

^1^H NMR spectra were recorded on a Bruker DPX‐400 spectrometer using deuterated solvent (materials section).

##### Size Exclusion Chromatography (SEC)

A Polymer Laboratories PL‐50 instrument equipped with differential refractive index (DRI) was used for SEC analysis. The system was fitted with 2× PLgel Mixed D columns (300 × 7.5 mm) and a PLgel 5 µm guard column. The eluent used was DMF with 0.1% LiBr. Samples were run at 1 mL min^−1^ at 50 °C. Poly(methyl methacrylate) standards (Agilent EasyVials) were used for calibration between 955,500 and 550 g mol^−1^. Analyte samples were filtered through a membrane with 0.22 µm pore size before injection. Experimental molar mass (Mn,SEC) and dispersity (Đ) values of synthesized polymers were determined by conventional calibration using Cirrus GPC software.

##### Dynamic Light Scattering

Dynamic light scattering (DLS) measurements were measured using a Malvern Zetasizer Nano ZS apparatus equipped with a He–Ne laser operated at λ = 633 nm and at a scattering angle of 173°. Particle size was measured at concentrations of 1 mg mL^−1^ in water at 25 °C, with three scans taken per measurement.

##### Transmission Electron Microscopy (TEM) Characterization

An aliquot (13 µL) of nanoparticles in water (1 mg mL^−1^) was deposited onto a Formvar carbon 200 mesh grid for DMA_150_–(HDD‐PIP)–DMA_150_ particles or a graphene oxide grid for NAM_150_–(HDD‐PIP)–NAM_150_ particles for 1 min and then the grid was blotted with filter paper (Fisherbrand, Grade 12). The sample was negatively stained with a 2% uranyl acetate solution in water (13 µL) for 1 min, and then the grid was again blotted with filter paper (Fisherbrand, Grade 12) and dried in air. Transmission electron microscopy analyses were carried out using a FEI Tecnai microscope using an accelerating voltage of 100 kV.

##### Theoretical Molar Mass Calculation

(1) $$M_{n,\;th}=M_{0}\;\frac{1+r}{\left(1+r-2rp\right)}$$

Equation 1: Calculation of theoretical number average molar mass (*M*_n,th_) according to Carothers equation, where *M*_0_ is the molar mass of the repeating polymer unit, *r* the stoichiometric ratio of diacrylate to amine and *p* the degree of polymerization which is assumed as 1. It is used to calculate the theoretical molar mass of PBAEs.

(2) $$M_{n,\;th}=\frac{{\left[M\right]}_{0}pM_{M}}{{\left[CTA\right]}_{0}}+M_{CTA}$$

Equation 2: Calculation of theoretical number average molar mass (*M*_n,th_) where [*M*]_0_ and [*CTA*]_0_ are the initial concentrations (in mol dm^−3^) of monomer and chain transfer agent respectively. *p* is the monomer conversion as determined by ^1^H NMR spectroscopy. *M*_M_ and *M*_CTA_ are the molar masses (g mol^−1^) of the monomer and chain transfer agent respectively. It is used to calculate the theoretical molar mass of the PBAE–RAFT ABA triblock copolymers.

#### 2.2.2 PBAE Synthesis

PBAEs were synthesized as previously reported.^[^ ^19^ ^]^ Butanediol diacrylate (5 g, 25.2 mmol) or hexanediol diacrylate (10 g, 44.2 mmol) was mixed with amine at a 1.1:1 molar ratio of monomer to amine in either DMSO (BDD‐3AP polymer) or dioxane (HDD‐PIP polymer) at 500 mg mL^−1^ and the reaction stirred in the dark at 90 °C for 24 h. Following reaction completion, the mixture was diluted (167 mg mL^−1^) and end‐capped using 2,2‐(ethylenedioxy)diethylamine (0.5 m) at 25 °C for 24 h. The resulting polymer was purified in tetrahydrofuran (THF), and diethyl ether (1:9) and the solvent removed under reduced pressure to yield a yellow, viscous liquid. Amine capping efficacy was assessed using ^1^H NMR with no acrylate peaks present following the capping steps. The final polymers were characterized by SEC and ^1^H NMR.

##### PBAE Functionalization with NHS‐PABTC

The selected PBAE (1 g, 1 eq.) and NHS‐PABTC (6 eq.) were solubilized in DMF (1 mg mL^−1^ final PBAE concentration) following which TEA (3 eq.) was added. The reaction was left to stir (450 rpm, 25 mm stirrer bar) in the dark at 25 °C for 48 h. Following reaction completion, the resulting PBAE–mCTAs were purified in THF and diethyl ether (1:9), and the solvent was removed under reduced pressure yielding the PBAE–mCTAs as yellow, viscous liquids. The final polymers were analyzed by SEC and ^1^H NMR.

#### 2.2.3 Grafting‐From RAFT Polymerization

RAFT polymer chain extension was conducted in dioxane under nitrogen with the selected monomers (1.5 m), selecting degrees of polymerization (DP) of 100, 200, and 300; using ACVA (10 mg mL^−1^ stock in dioxane) as the initiator and keeping the CTA to initiator ratio as 2. The reaction was left to stir under nitrogen at 70 °C for 24 h. The resulting polymers were analyzed using ^1^H NMR and SEC. Conversion was assessed using ^1^H NMR by comparing the integration of the acrylate/acrylamide peaks before and after reaction completion, using the PBAE polyester protons a 4.01 ppm as a reference. Rhodamine‐tagged polymers were synthesized by adding an acryloxyethyl thiocarbamoyl rhodamine B stock solution in DMF (10 µg mL^−1^) to the reaction mixture, targeting a 0.1% molar dye content in total number of monomer moles used.

#### 2.2.4 PBAE–RAFT Particle Formulation

##### Micelle Formulation by Solvent Precipitation

Micelles were formulated by adding the polymer solution (100 µL, 10 mg mL^−1^) in acetone or THF to Mili‐Q grade water (1 mL) in a scintillation vial (20 mL) at a rate of 0.1 mL min^−1^ under constant stirring (960 rpm, 25 mm stirrer bar) to yield a final polymer concentration of 1 mg mL^−1^. The suspension was left to stir for 1 h following which the organic solvent was removed under reduced pressure.

##### Micelle Formulation by Direct Water Solubilization

Micelles were formulated by adding Mili‐Q grade water (5 mL) to dry polymer (5 mg) in a scintillation vial (20 mL), under constant stirring (960 rpm, 25 mm stirrer bar) and suspension being left to stir for 1 h, to yield a final polymer concentration of 1 mg mL^−1^.

#### 2.2.5 Drug Encapsulation

Mili‐Q grade water (5 mL) and a docetaxel (DTX) solution in dimethyl sulfoxide (DMSO) (0.5 mL, 5 mg mL^−1^) were simultaneously added to weighed out polymer (5 mg) and left to stir (960 rpm, 25 mm stirrer bar) for 2 h to yield a final polymer concentration of 1 mg mL^−1^. Centrifugal filtration (3500 Dalton molecular weight cutoff, Amicon) was applied to purify the unencapsulated drug. The experiments were repeated three times, each time using three technical replicates.

##### Quantification of Drug Load by HPLC

Drug‐loaded particles (5 mL) were freeze‐dried for 24 h (0.98 mbar), dissolved in a 50:50 mixture of DMSO and trifluoroacetic acid (TFA), and left to stir for 3 h. The solution was then diluted 1:10 in DMSO and encapsulation levels were assessed by high performance liquid chromatography (HPLC) (Agilent Technologies 1200 series, USA). The experiment was repeated three times, each time using three technical replicates.

Drug loading of DTX was assessed using a C18 (4.6 × 250 mm; 5 µm) analytical column (ZORBAX Eclipse Plus). The UV detector was operated at 239 nm. The mobile phase consisted of a mixture of 0.1% orthophosphoric acid aqueous solution and ACN (40:60, v/v). The flow rate was set at 1.0 mL min^−1^ and injection volume at 25 µL.^[^ ^20^ ^]^

Drug loading and encapsulation efficiency were calculated using the following equations:

(3) $$\mathrm{Drug}\;\mathrm{loading}\left(\%\right)=\frac{\mathrm{Weight}\;\mathrm{of}\;\mathrm{loaded}\;\mathrm{drug}}{\mathrm{Total}\;\mathrm{weight}\;\mathrm{of}\;\mathrm{polymer}}\times 100$$

Equation 3: Calculation of percentage drug load.

(4) $$\begin{array}{ccc} & & \mathrm{Drug}\;\mathrm{encapsulation}\;\%\left(\frac{w}{w}\right)\\ & & \;=\frac{\mathrm{Total}\;\mathrm{amount}\;\mathrm{of}\;\mathrm{drug}-\mathrm{unloaded}\;\mathrm{drug}}{\mathrm{Total}\;\mathrm{amount}\;\mathrm{of}\;\mathrm{drug}}\times 100 \end{array}$$

Equation 4: Calculation of percentage drug encapsulation.

#### 2.2.6 Docetaxel Release Study

DTX release from polymer carrier was assessed in phosphate buffer at pH = 7.4, containing 0.25% Tween 20. A sample (2.5 mL, 2 mg mL^−1^) of DTX‐loaded nanoparticles was placed in a dialysis membrane (3500 Dalton molecular weight cutoff, Spectrum Labs) The micellar solution was dialyzed against 30 mL of release media at 37 °C, over 72 h, and samples (1 mL) were collected at appropriate timepoints and replaced with the same amount of fresh medium to maintain sink conditions. DTX quantification from the release samples was conducted by HPLC as described above.

#### 2.2.7 Cell Culture of MDA‐MB‐231 Cells

The MDA‐MB‐231 triple‐negative breast cancer cell lines were obtained from the American Type Culture Collection (Manassas, VA). These cell lines were cultured in Roswell Park Memorial Institute (RPMI) medium supplemented with 10% (v/v) fetal bovine serum (FBS) and 2 mm l‐glutamine. The cultures were incubated at 37 °C in a 5% CO_2_ environment. When the cell monolayers reached 80% confluency, the cancer cells were detached using 1× Trypsin–EDTA solution (Sigma–Aldrich Co., St. Louis, MI, USA). To determine the live cell number, an aliquot from the cell suspension was stained with Trypan blue (Sigma–Aldrich Co., St. Louis, MI, USA) under an optical microscope. This staining allowed for the identification of dead cells, while the remaining unstained cells were counted using a hemocytometer.

#### 2.2.8 Uptake in 2D Monolayers of MDA‐MB‐231 Cells

MDA‐MB‐231 cells were seeded in CellView 35 mm diameter glass bottom cell culture dishes at a density of 2.5 × 10^5^ cells per dish and cultured for 24 h in RPMI. Then, the media was removed, and 50 µg mL^−1^ rhodamine‐labeled nanoparticles were added and incubated with cells for 4 h at 37 °C with 5% CO_2_. Following exposure, nanoparticle solutions were removed, and cells were washed three times with ice‐cold phosphate‐buffered saline (PBS). Cells were then stained with 10 µg mL^−1^ Hoechst 33342 (Thermo‐Fisher) for 15 min and washed three times with ice‐cold PBS. Afterwards, 75 nm LumiTracker Lyso Green (Lumiprobe) was applied in PBS for 30 min before the staining solution was finally removed and the cells washed twice with PBS. Subsequently, the cells were imaged using Leica TCS SPE laser scanning confocal microscope. The images were processed using ImageJ software and the JACoP (Just Another Colocalisation Plugin) in ImageJ was used for the calculation of Pearson's correlation coefficient for co‐localization studies.^[^ ^21^ ^]^

#### 2.2.9 Cytotoxic Activity in 2D Monolayers of MDA‐MB‐231 Cells

MDA‐MB‐231 cells were seeded in tissue culture treated Thermo Scientific Nunc MicroWell 96‐Well Optical‐Bottom black plates at a density of 1 × 10^4^ cells per well and cultured for 24 h in RPMI. The media was then removed, and the cells treated with different concentrations of blank NAM_150_–(HDD‐PIP)–NAM_150_ and DMA_150_–(HDD‐PIP)–DMA_150_ nanoparticles for 72 h. Subsequently, the treatments were replaced with 100 µL of 10% PrestoBlue HS Cell Viability Reagent in RPMI and incubated for 20 min. The fluorescence intensity was measured using an excitation/emission wavelength of 544/590 nm on a FLUOstar Omega plate reader (BMG LABTECH, UK).

## 3 Results and Discussion

### 3.1 Development of Synthetic Methodology

**Figure 1:**
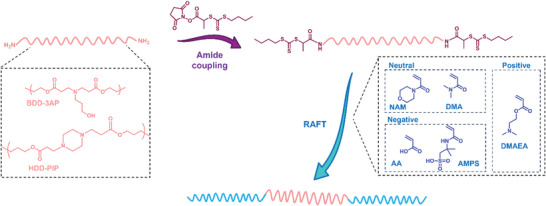
Outline of the synthetic methodology developed to make PBAE–RAFT triblock copolymers, including the structural details of the core PBAE scaffolds used and RAFT comonomers tested.

To develop our grafting‐from methodology we first synthesized two PBAE models via traditional one‐pot aza‐Michael addition chemistry. A hydrophilic butanediol diacrylate (BDD)‐ 3‐amino propan‐1‐ol (3AP) copolymer, and a hydrophobic hexanediol diacrylate (HDD)‐ piperazine (PIP) copolymer utilizing 1.1:1 diacrylate:amine ratio, using previously reported conditions.^[^ ^19^ ^]^ Both polymers displayed molar masses close to the theoretical value calculated via Carothers equation and exhibited residual acrylate signals in the ^1^H NMR spectra (**Table** **1**). The terminal acrylates were then end‐capped with an excess of (2,2‐(ethylenedioxy)diethylamine) to avoid any PBAE coupling. The final amino functional PBAEs displayed similar molar masses to their acrylate terminated analogs while full amine functionalization was confirmed by the complete disappearance of the acrylate signals in the ^1^H NMR spectra. For the HDD–PIP polymer, we observed an increase in molar mass following polymer purification in a mixture of THF and cold ether (1:9), hypothesized to be caused by the solubility of lower molar mass chains in the purification solvent leading to their removal during this step. The amino terminated hydrophobic and hydrophilic PBAEs were then further functionalized at the α and ω end groups with an *N*‐hydroxysuccinimide functional RAFT agent, NHS–PABTC, to form PBAE macromolecular chain transfer agents (PBAE–mCTA) (**Figure** **1**; Figure S1, Supporting Information). Following CTA functionalization, we observed a further increase in *M*_n,SEC_, particularly for the HDD–PIP polymer, hypothesized to originate from the solubilization of lower molar mass chains in the purification solvent.

**Table 1:** Molar mass characterization of PBAE and PBAE–mCTAs. Polymer ^1^H NMR and SEC characterization is available in Figures S2 and S3 (Supporting Information).

Polymer	*M_n_*_, th_ [g mol^−1^]^a^ ^)^	*M*_n, SEC_ [g mol^−1^]^b^ ^)^	*Ð*
BDD‐3AP	5800	6000	1.40
BDD‐3AP‐mCTA	6302	9200	1.72
HDD‐PIP	6700	14000	1.43
HDD‐PIP‐mCTA	7200	18000	1.37

^a)^ Calculated using Equation 1;

^b)^ Determined using DMF–SEC.

**Figure 2:**
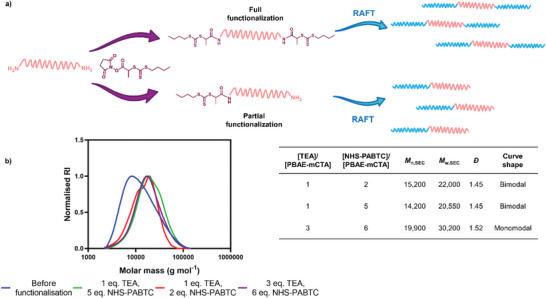
Optimization of synthetic methodology used to synthesize the PBAE–mCTA, based on NAM_50_–(BDD‐3AP)–NAM_50_ molar mass distributions (g mol^−1^), where a) outline of full and partial PBAE functionalization with NHS‐PABTC and the resulting copolymer structures; b) molar mass distributions of NAM_50_–(BDD‐3AP)–NAM_50_ defined by *M*_n,SEC_ where final polymer was synthesized using 1 eq. of TEA and 2 eq. of NHS–PABTC (red); 1 eq. of TEA and 5 eq. of NHS–PABTC (green); and 3 eq. of TEA and 6 eq. of NHS‐PABTC (purple); Inset table contains the comparison of final NAM_50_‐(BDD‐3AP)‐NAM_50_ polymer number average molar mass (*M*_n_), weight average molar mass (*M*_w_), polydispersity (*Ð*), and curve shape depending on TEA and NHS‐PABTC equivalents used to synthesize the starting PBAE–mCTA.

In order to produce triblock copolymers efficiently via our grafting‐to methodology, it was imperative to ensure the PBAE–mCTAs expressed RAFT agents at both ends on all PBAE molecules, as any proportion of monofunctionalized PBAE–mCTA would result in bimodal molar mass distribution for the resultant triblock copolymers (**Figure** **2**a). To optimize this process, we tested different PBAE:NHS–PABTC equivalents (2–6 eq.) and PBAE:triethylamine equivalents (0–3 eq) for this reaction. Unfortunately, NHS–PABTC attachment was not easily detectable via ^1^H NMR spectroscopy, hence to assess functionalization efficiency we postulated that the shape of the molar mass distribution following RAFT polymerization would illustrate the relative functionalization efficiency. We observed that grafts with DP100 *N*‐acryloylmorpholine (NAM) from the BDD‐3AP‐mCTA PBAE produced with 6 eq. NHS–PABTC and 3 eq. TEA yielded monomodal NAM_50_–(BDD‐3AP)–NAM_50_ ABA ternary graft copolymers suggesting complete functionalization on both end groups. In contrast, graft copolymers prepared with <5 eq. NHS–PABTC and <3 eq. TEA yielded bimodal molar mass distributions suggesting incomplete RAFT agent functionalization (Figure 2b). PBAE functionalization was further verified by *M*_n,SEC_ with an increase from 9500 Da for BDD‐3AP‐mCTA to masses of ≈20 000 for the partially functionalized polymers and an *M*_n,SEC_ of 30 200 for fully functionalized NAM_50_‐(BDD‐3AP)‐NAM_50_. We therefore proceeded with 6 eq. of TEA and 3 eq. of NHS–RAFT as the selected method parameters. Kinetic analysis of the polymerization of BDD‐3AP‐mCTA and HDD–PIP–mCTA with the NAM monomer showed over 90% monomer conversion was achieved within 4 h with a gradual increase in molar mass observed (Figures S2 and S3, Supporting Information).

Following the optimization of the grafting‐from methodology we sought to explore the synthetic versatility of the platform by expanding the monomer set for the RAFT polymerization step. Accordingly, we employed a range of five vinyl monomers, two neutral charge monomers (NAM and dimethylacrylamide (DMA)), two negatively charged monomers (2‐acrylamidopropanesulfonate (AMPS) and acrylic acid (AA)), and a positively charged monomer (*N*,*N′*‐dimethylaminoacrylate (DMAEA)), to extend both HDD–PIP and BDD‐3AP PBAE‐mCTAs, targeting a DP of 100 at both ends of the PBAE–mCTA in each case (Table 2). We found the RAFT polymerization only achieved high conversion rates (>90%) for neutral monomers, with the negative and positively charged monomers yielding low monomer conversion. We expect this may be due to poor solubility of the AMPS monomer in solvents appropriate for the PBAE–mCTAs and acidic degradation of the PBAE–mCTAs by the AA and AMPS monomers, verified by ^1^H NMR spectroscopy showing the disappearance of the polyester signal present at around 4 ppm following HDD–PIP–mCTA RAFT with AA and BDD‐3AP‐mCTA RAFT with AMPS (Figures S4 and S6, Supporting Information).

**Table 2:** Characterization of PBAE–RAFT ABA‐triblock copolymers. Polymer ^1^H NMR and SEC characterization is available in Figures S3 and S4 (Supporting Information).

Polymer	DP_target_	Conversion %	*M*_n, th_ [g mol^−1^]^a^ ^)^	*M*_n, SEC_ [g mol^−1^]^b^ ^)^	*Ð*
NAM_50_‐(BDD‐3AP)‐NAM_50_	100	100%	23 600	20 000	1.52
DMA_50_‐(BDD‐3AP)‐DMA_50_	100	98%	19 200	18 000	1.25
AA_50_‐(BDD‐3AP)‐AA_50_	100	78%	N/A	N/A	N/A
AMPS_50_‐(BDD‐3AP)‐AMPS_50_	100	PBAE degradation	N/A	N/A	N/A
DMAEA_50_‐(BDD‐3AP)‐DMAEA_50_	100	14%	N/A	N/A	N/A
NAM_50_‐(HDD‐PIP)‐NAM_50_	100	91%	30 800	28 000	1.74
DMA_50_‐(HDD‐PIP)‐DMA_50_	100	97%	27 600	27 000	1.64
AA_50_‐(HDD‐PIP)‐AA_50_	100	PBAE degradation	N/A	N/A	N/A
AMPS_50_‐(HDD‐PIP)‐AMPS_50_	100	23%	N/A	N/A	N/A
DMAEA_50_‐(HDD‐PIP)‐DMAEA_50_	100	41%	N/A	N/A	N/A
NAM_100_‐(HDD‐PIP)‐NAM_100_	200	99%	45 900	33 000	1.84
DMA_100_‐(HDD‐PIP)‐DMA_100_	200	98%	37 400	33 000	1.94
NAM_150_–(HDD‐PIP)–NAM_150_	300	99%	59 900	39 000	1.94
DMA_150_–(HDD‐PIP)–DMA_150_	300	98%	47 100	39 000	1.92

^a)^ Calculated using Equation 2;

^b)^ Determined using DMF–SEC.

**Figure 3:**
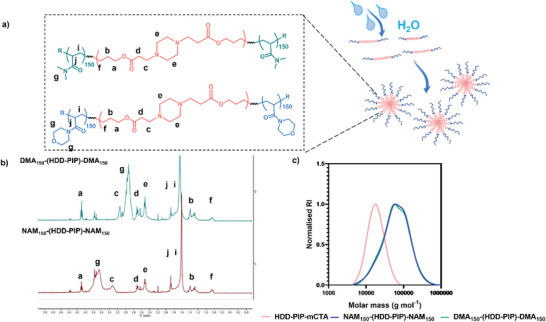
Polymer characterization of NAM_150_–(HDD‐PIP)–NAM_150_ and DMA_150_–(HDD‐PIP)–DMA_150_: a) Structures of both polymers; b) ^1^H NMR spectra in CDCl_3_; **c)** Molar mass distribution (g mol^−1^) of the obtained polymers and the starting PBAE–mCTA;.

Another key advantage of our grafting‐from process is that the RAFT polymerization step enables the precise control of the graft lengths and therefore control over final copolymer molecular mass. To exemplify this, we performed graft‐copolymerizations with both DMA and NAM monomers at DP100, 200, and 300 by varying the (vinyl monomer)/(PABTC–mCTA) ratio (**Table** **2**), (Figures 2a,b). All polymerizations achieved conversions above 90% with SEC chromatograms exhibiting higher molar mass polymers with increasing target chain length (**Figure** **3**c; Figure S5, Supporting Information).

### 3.2 Assembly of Polymeric Nanoparticles

We have demonstrated the ability to produce a range of triblock graft copolymers utilizing the PBAE–mCTA platform. Given the extensive use of PBAEs as biomaterials, we hypothesized that such block copolymers may have potential as a drug delivery platform. Given their hydrophobic PBAE central block and hydrophilic coronas, we initially tested the ability of NAM_50_–(BDD‐3AP)–NAM_50_, DMA_50_–(BDD‐3AP)–DMA_50_, NAM_50_–(HDD‐PIP)–NAM_50_, and DMA_50_–(HDD‐PIP)–DMA_50_ copolymers to form block copolymer micelle assemblies. Micelle formation was conducted through a solvent precipitation methodology, using tetrahydrofuran or acetone as the organic solvent applied to solubilize the polymer, followed by its dropwise addition to stirring water and subsequent removal of organic solvent by evaporation. It was determined that polymers based on a BDD‐3AP core were not suitable for particle assembly, with the polymer either not forming particles (DMA copolymer) or forming unstable particles (NAM copolymer). Stability was assessed through particle addition to ionic media (phosphate buffer saline at pH 7.4), with a rapid aggregation of the NAM_50_–(BDD‐3AP)–NAM_50_ particles observed, confirmed by an increase in particle diameter from below 200 nm to above 1000 nm.

Meanwhile block copolymers derived from the HDD–PIP PBAE–mCTA produced particles with mean diameters of 207 and 227 nm for NAM_50_–(HDD‐PIP)–NAM_50_ and DMA_50_–(HDD‐PIP)–DMA_50_, respectively, which showed no observable aggregation when diluted in PBS (Figure S7, Supporting Information). For both particle types, the sample polydispersity (PDi) was below 0.3, with NAM_50_‐(HDD‐PIP)‐NAM_50_ showing a mean PDi of 0.19 and DMA_50_–(HDD‐PIP)–DMA_50_ NPs a mean PDi of 0.25. A monomodal particle size distribution by intensity was observed for each particle type. We hypothesized that this enhanced stability was due to the greater hydrophobicity of HDD–PIP, compared to BDD‐3AP, enhancing the nonpolar interactions and thus stabilizing the resulting particles.

**Figure 4:**
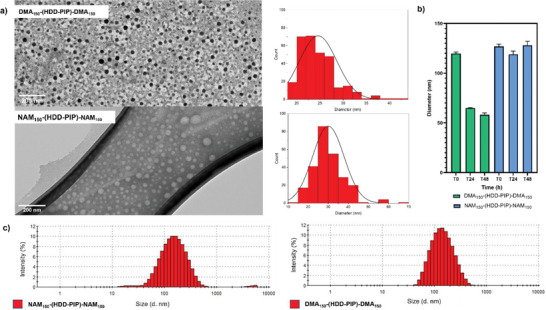
Particle characterization of NAM_150_–(HDD‐PIP)–NAM_150_ and DMA_150_–(HDD‐PIP)–DMA_150_: a) TEM particle size distribution (nm) and TEM images of uranyl acetate‐stained NPs on Formvar carbon 200 mesh grid for DMA_150_–(HDD‐PIP)–DMA_150_ particles or a graphene oxide grid for NAM_150_–(HDD‐PIP)–NAM_150_ particles, scale bar at 200 nm; b) Particle diameter (nm) across 48 h, at room temperature, in water at 1 mg mL^−1^; c) Particle size (nm) distribution by intensity (%) in water, at room temperature, at 1 mg mL^−1^.

We sought to optimize this formulation process to produce smaller nanoparticles, ideally below 200 nm as these have been shown to have improved uptake to tumor cells and enhanced biofilm penetration, two potential applications for these materials.^[^ ^22^, ^23^, ^24^ ^]^ Furthermore, eliminating the use of organic solvents may also improve the translation of these materials.^[^ ^25^ ^]^ We therefore sought to solubilize directly the prepared HDD–PIP polymers in water without the use of organic solvent (Figure 3a). This was performed through direct water addition to dry polymer powder, followed by stirring for 1 h. We found that while for the NAM_50_–(HDD‐PIP)–NAM_50_ and DMA_50_–(HDD‐PIP)–DMA_50_ polymers direct assembly was not possible, for NAM_100_–(HDD‐PIP)–NAM_100_, NAM_150_–(HDD‐PIP)–NAM_150_, DMA_100_–(HDD‐PIP)–DMA_100_, and DMA_150_–(HDD‐PIP)–DMA_150_ polymers, we achieved spontaneous particle assembly following water addition. We hypothesized this result from the longer hydrophilic RAFT chains in the latter promoting polymer water solubility and particle assembly. The diameters of the particles obtained through direct polymer solubilization, were in each case below 200 nm, with a mean diameter of 127 nm reported for NAM_150_–(HDD‐PIP)–NAM_150_ and 120 nm for DMA_150_–(HDD‐PIP)–DMA_150_ (**Figure** **4**b). Sample PDI were 0.26 and 0.24 for NAM_150_–(HDD‐PIP)–NAM_150_ and DMA_150_–(HDD‐PIP)–DMA_150_, respectively. DMA_150_–(HDD‐PIP)–DMA_150_ particles gave a monomodal size distribution by intensity, ranging from 40 to 460 nm, while for NAM_150_–(HDD‐PIP)–NAM_150_ NPs two particle populations were observed, one ranging from 10 to 40 nm and the other from 40 to 500 nm (Figure 4c). TEM images provided further evidence the diameters for both the polymer variants were below 200 nm (Figure 4a).

Given the potential application of these materials in a biomedical context we assessed particle stability in water, at room temperature across 48 h, to evaluate whether particle aggregation occurs. DMA_150_–(HDD‐PIP)–DMA_150_ showed a decrease of mean diameter from 120 to 56 nm, while NAM_150_–(HDD‐PIP)–NAM_150_ particles retained a mean diameter of 128 nm throughout the stability study (Figure 4b). For each NP type, a decrease in particle PDI was observed (from 0.26 to 0.13 and from 0.24 to 0.17 for NAM_150_–(HDD‐PIP)–NAM_150_ and DMA_150_–(HDD‐PIP)–DMA_150_, respectively). For both types of polymer particles, aggregation was not observed. The reduction in particle size for the DMA_150_–(HDD‐PIP)–DMA_150_ triblock copolymer may be due to the particles initially resolving in a kinetically trapped state and slowly reconfiguring into smaller nanoparticles during the course of the stability study.^[^ ^26^ ^]^

### 3.3 Drug Encapsulation and In Vitro Release

We next evaluated the potential of NAM_150_–(HDD‐PIP)–NAM_150_ and DMA_150_–(HDD‐PIP)–DMA_150_ micelles as delivery vehicles for hydrophobic drug molecules. Docetaxel (DTX) was selected as the hydrophobic drug of choice due to its low solubility in water and bulky polycyclic structure, combined with high activity in most cancer cell lines.^[^ ^27^ ^]^

**Figure 5:**
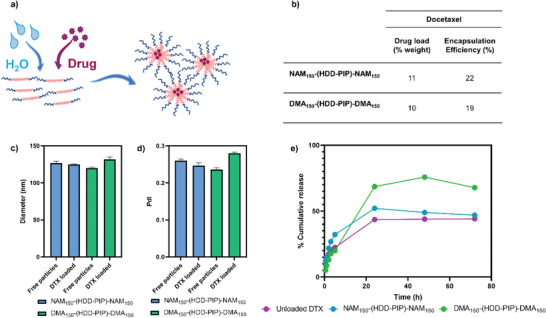
Drug encapsulation in NAM_150_–(HDD‐PIP)–NAM_150_ and DMA_150_–(HDD‐PIP)–DMA_150_ particles, where: a) Scheme of DTX loading in PBAE–RAFT particles b) Inset table that describes cumulative drug load (%) and encapsulation efficiency of DTX, defined by HPLC; c) Change in mean particle diameter (nm) following DTX encapsulation, in water at 1 mg mL^−1^ where average diameter for DTX loaded particles is 125 nm for NAM_150_–(HDD‐PIP)–NAM_150_ and 132 nm DMA_150_–(HDD‐PIP)–DMA_150_; d) Change in particle PdI following DTX encapsulation, in water at 1 mg mL^−1^ where average PdI for NAM_150_–(HDD‐PIP)–NAM_150_ is 0.26 and 0.24 for DMA_150_–(HDD‐PIP)–DMA_150_ for DTX loaded particles is 0.25 for NAM_150_–(HDD‐PIP)–NAM_150_ and 0.28 DMA_150_–(HDD‐PIP)–DMA_150_; e) DTX release from NAM_150_–(HDD‐PIP)–NAM_150_ (blue) and DMA_150_–(HDD‐PIP)–DMA_150_ (green) particles and unloaded DTX (purple) in phosphate buffer pH 7.4, containing 0.25% Tween 20, at 37 ^°^C, across 72 h, samples analyzed by HPLC.

DTX encapsulation was achieved through simultaneous addition of water and a drug solution in DMSO (**Figure** **5**a), to weighed out dry polymer, followed by stirring for 2 h and subsequent removal of unencapsulated DTX by centrifugal filtration. This yielded a mean drug load of 10% ± 2.6% and 11% ± 2.8% for DMA_150_–(HDD‐PIP)–DMA_150_ and NAM_150_–(HDD‐PIP)–NAM_150_, respectively, across three experimental replicates, demonstrating polymer suitability for hydrophobic drug encapsulation (Figure 5b). A negligible change in mean particle diameter and polydispersity following DTX encapsulation was observed for both particle variants tested (Figure 5c,d). The findings suggest our block copolymer micelle assemblies are suitable for the delivery of hydrophobic drug molecules.

In vitro release of DTX from NAM_150_–(HDD‐PIP)–NAM_150_ and DMA_150_–(HDD‐PIP)–DMA_150_ micelles was evaluated in phosphate buffer at pH 7.4 (blood pH) simulating body temperature (37 °C; Figure 5e). NAM_150_–(HDD‐PIP)–NAM_150_ particles achieved 32% drug release within the first 5 h, reaching 52% following 24 h. DTX release from DMA_150_–(HDD‐PIP)–DMA_150_ micelles was initially slower, reaching 20% after 5 h followed by an increase to 69% after 24 h. Following 48 h DTX release from DMA_150_–(HDD‐PIP)–DMA_150_ increased to 76%, followed by a reduction in the percentage of cumulative release to 68% following 72 h, hypothesized to originate from the hydrolysis of DTX ester bonds. A drop in DTX cumulative release from the NAM_150_–(HDD‐PIP)–NAM_150_ polymer was also observed with a reduction from 52% observed after 24 h to 47% at 72 h. Comparatively, the free drug control achieved only 44% cumulative drug release following 24 and 72 h due to the limited solubility of DTX. Following DTX encapsulation in both NAM_150_–(HDD‐PIP)–NAM_150_ and DMA_150_–(HDD‐PIP)–DMA_150_ NPs an improvement in drug solubility was observed, stipulated to originate from the nano size of the particles influencing the solubility and dissolution rate of DTX through decreased particle size and increased surface area, as described by the Kevin and Noyes–Whitney equations.^[^ ^28^, ^29^ ^]^

### 3.4 Uptake in 2D Monolayers of MDA‐MB‐231 Breast Cancer Cells

**Figure 6:**
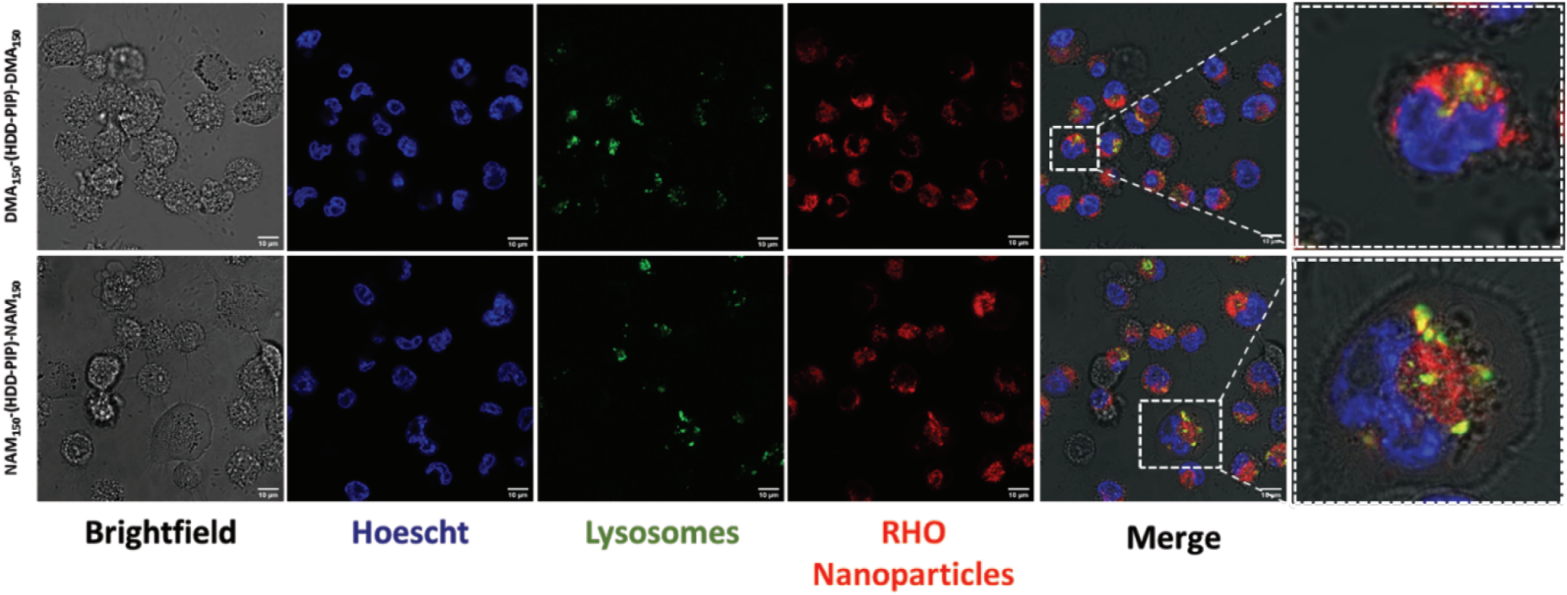
Nanoparticle uptake studies: a) Confocal microscopy imaging at 63× illustrating the cellular uptake of rhodamine labelled NAM_150_–(HDD‐PIP)–NAM_150_ and DMA_150_–(HDD‐PIP)–DMA_150_ nanoparticles in MDA‐MB‐31 breast cancer cell lines and their co‐localization within lysosomes (LumiTracker Lyso Green). Scale bar = 10 µm. The image contrast was enhanced through saturating the pixels by 0.1% using the “enhance contrast” feature in ImageJ across all channels and the original images attached in Figure S8 (Supporting Information).

Effective drug delivery systems are pivotal in enhancing therapeutic outcomes in cancer treatment,^[^ ^30^ ^]^ with nanomaterials emerging as promising candidates for targeted drug delivery in cancer therapy.^[^ ^31^ ^]^ Hence, we investigated the cellular uptake and lysosomal co‐localization of rhodamine functionalized NAM_150_–(HDD‐PIP)–NAM_150_ and DMA_150_–(HDD‐PIP)–DMA_150_ NPs in MDA‐MB‐231 breast cancer cells. Successful cellular uptake and preferential localization in lysosomes are crucial factors for ensuring efficient drug delivery and effective treatment outcomes.^[^ ^31^, ^32^ ^]^ Our results demonstrated a robust cellular uptake of the nanoparticles within breast cancer cells. Confocal microscopy revealed a clear distribution of nanoparticles throughout the cytoplasm, confirming their successful internalization as illustrated in **Figure** **6**. Furthermore, the assessment of co‐localization demonstrated a significant degree of overlap between the nanoparticles and lysosomes, with a Pearson correlation coefficient of 0.5 for each nanoparticle system. This finding indicates that a substantial proportion of the NPs are trafficked to the lysosomes, suggesting their potential utilization as templates for drug delivery, considering lysosomes are key organelles involved in nanoparticle degradation and effective release of the payload.^[^ ^33^ ^]^ This promising result opens avenues for future studies to explore the use of these copolymers for the delivery of a range of chemotherapeutic agents and further elaborate on the precise mechanisms underlying the lysosomal accumulation and subsequent release of drugs from these NPs.

### 3.5 Cytotoxicity Assessment of Nanoparticles in 2D Monolayers of MDA‐MB‐231 Breast Cancer Cells

The cytotoxic activity of NAM_150_–(HDD‐PIP)–NAM_150_ and DMA_150_–(HDD‐PIP)–DMA_150_ were assessed in vitro on MDA‐MB‐231 breast cancer cells using the PrestoBlue Cell Viability Reagent. Both particles demonstrated cellular viability above 80% across all concentrations, indicating the biocompatibility of these polymers when used with cells at the tested concentrations (Figure S9, Supporting Information). Our findings suggest that these NPs could serve as potential platforms for loading chemotherapeutic agents, allowing for the management of various cancer types.

## 4 Conclusion

We report a new grafting‐from methodology to functionalize PBAEs with neutrally charged RAFT monomers, to obtain ABA tri‐block copolymers at a range of DPs and with monomodal molar mass distributions. We demonstrate successful NP assembly of selected materials, NAM_150_–(HDD‐PIP)–NAM_150_ and DMA_150_–(HDD‐PIP)–DMA_150_, obtaining particle sizes below 200 nm and achieving over 10% drug loading of the model hydrophobic drug, DTX. Rhodamine tagged NAM_150_–(HDD‐PIP)–NAM_150_ and DMA_150_–(HDD‐PIP)–DMA_150_ particles were shown to be successfully internalized in MDA‐MB‐231 breast cancer cells, particularly in lysosomes, demonstrating a potential application as vehicles for therapeutic drug delivery.

Our findings facilitate the preparation of structurally diverse ABA tri‐block PBAE–RAFT copolymers, therefore expanding the scope of PBAEs and introducing a new class of biomaterial. Considering the plethora of neutral acrylate and acrylamide monomers that can be attached through the RAFT process, copolymer properties can now be finely tuned to tailor them for selected applications. Moreover, precise control over the final RAFT polymer DPs may offer bespoke ways of controlling end polymer properties, facilitating NP design to tailor drug release profiles, enhance stability in physiological fluids, and target specific cell types and environments. Furthermore, considering the successful encapsulation of DTX, the developed polymeric micelles can be applied for the oral delivery of a range of hydrophobic drugs, by enhancing their bioavailability, through improvement of solubility, permeability, and dissolution rate. This can be particularly expanded to deliver drug combination therapies by achieving enhanced therapeutic efficacy.

## Conflict of Interest

The authors declare no conflict of interest.

## Supporting information

Supporting Information

## Data Availability

The data that support the findings of this study are available from the corresponding author upon reasonable request.

## References

[1] D. M. Lynn , R. Langer , J. Am. Chem. Soc. 2000, 122, 10761.

[2] Y. Liu , Y. Li , D. Keskin , L. Shi , Adv. Healthcare Mater. 2019, 8, e1801359. 10.1002/adhm.201801359 30549448

[3] Y. Liu , H. J. Busscher , B. Zhao , Y. Li , Z. Zhang , H. C. Van Der Mei , Y. Ren , L. Shi , ACS Nano 2016, 10, 4779. 26998731 10.1021/acsnano.6b01370

[4] J. J. Green , R. Langer , D. G. Anderson , Acc. Chem. Res. 2008, 41, 749. 18507402 10.1021/ar7002336 PMC3490629

[5] X. Su , J. Fricke , D. G. Kavanagh , D. J. Irvine , Mol. Pharm. 2011, 8, 774. 21417235 10.1021/mp100390w PMC3354687

[6] J. Wei , L. Zhu , Q. Lu , G. Li , Y. Zhou , Y. Yang , L. Zhang , J. Control. Release 2023, 354, 337. 36623697 10.1016/j.jconrel.2023.01.002

[7] Y. Zhang , R. Wang , Y. Hua , R. Baumgartner , J. Cheng , ACS Macro Lett. 2014, 3, 693. 35590770 10.1021/mz500277j

[8] J. Karlsson , K. R. Rhodes , J. J. Green , S. Y. Tzeng , Expert Opin. Drug. Deliv. 2020, 17, 1395. 32700581 10.1080/17425247.2020.1796628 PMC7658038

[9] C. Zhang , T. An , D. Wang , G. Wan , M. Zhang , H. Wang , S. Zhang , R. Li , X. Yang , Y. Wang , J. Control. Release 2016, 226, 193. 26896737 10.1016/j.jconrel.2016.02.030

[10] J. Kim , S. K. Mondal , S. Y. Tzeng , Y. Rui , R. Al‐Kharboosh , K. K. Kozielski , A. G. Bhargav , C. A. Garcia , A. Quiñones‐Hinojosa , J. J. Green , ACS Biomater. Sci. Eng. 2020, 6, 2943. 33463272 10.1021/acsbiomaterials.0c00116 PMC8035708

[11] J. Kim , Y. Kang , S. Y. Tzeng , J. J. Green , Acta Biomater. 2016, 41, 293. 27262740 10.1016/j.actbio.2016.05.040 PMC6061916

[12] T. Thambi , V. H. Giang Phan , S. H. Kim , T. M. Duy Le , H. T. T. Duong , D. S. Lee , Biomater. Sci. 2019, 7, 5424. 31638108 10.1039/c9bm01161g

[13] R. A. Cordeiro , D. Farinha , N. Rocha , A. C. Serra , H. Faneca , J. F. J. Coelho , Macromol. Biosci. 2015, 15, 215. 25399846 10.1002/mabi.201400424

[14] S. C. Larnaudie , J. C. Brendel , K. A. Jolliffe , S. Perrier , J. Polym. Sci. A Polym. Chem. 2016, 54, 1003.

[15] D. Roy , J. T. Guthrie , S. Perrier , Macromolecules 2005, 38, 10363.

[16] P. De , M. Li , S. R. Gondi , B. S. Sumerlin , J. Am. Chem. Soc. 2008, 130, 11288. 18665597 10.1021/ja804495v

[17] G. Moad , Y. K. Chong , A. Postma , E. Rizzardo , S. H. Thang , Polymer 2005, 46, 8458.

[18] C. J. Ferguson , R. J. Hughes , B. T. T. Pham , B. S. Hawkett , R. G. Gilbert , A. K. Serelis , C. H. Such , Macromolecules 2002, 35, 9243.

[19] N. S. Bhise , R. S. Gray , J. C. Sunshine , S. Htet , A. J. Ewald , J. J. Green , Biomaterials 2010, 31, 8088. 20674001 10.1016/j.biomaterials.2010.07.023 PMC3175420

[20] P. S. Kumbhar , S. K. Diwate , U. G. Mali , T. U. Shinde , J. I. Disouza , A. S. Manjappa , Ann. Pharm. Fr. 2020, 78, 398. 32681903 10.1016/j.pharma.2020.07.004

[21] S. Bolte , F. P. Cordelières , J. Microsc. 2006, 224, 213. 17210054 10.1111/j.1365-2818.2006.01706.x

[22] Y. H. Bae , K. Park , J. Control. Release 2011, 153, 198. 21663778 10.1016/j.jconrel.2011.06.001 PMC3272876

[23] S. A. Kulkarni , S.‐S. Feng , Pharm. Res. 2013, 30, 2512. 23314933 10.1007/s11095-012-0958-3

[24] K. Forier , A.‐S. Messiaen , K. Raemdonck , H. Nelis , S. De Smedt , J. Demeester , T. Coenye , K. Braeckmans , J. Control. Release 2014, 195, 21. 25125326 10.1016/j.jconrel.2014.07.061

[25] M. R. Mozafari , Cell Mol. Biol. Lett. 2005, 10, 711. 16341279

[26] Y. Wang , J. He , C. Liu , W. H. Chong , H. Chen , Angew. Chem., Int. Ed. 2015, 54, 2022. 10.1002/anie.201402986 25536948

[27] Y. Mi , Y. Liu , S.‐S. Feng , Biomaterials 2011, 32, 4058. 21396707 10.1016/j.biomaterials.2011.02.022

[28] A. A. Noyes , W. R. Whitney , J. Am. Chem. Soc. 1897, 19, 930.

[29] K. G. Nelson , J. Pharm. Sci. 1972, 61, 479. 5013396 10.1002/jps.2600610340

[30] R. S. Riley , C. H. June , R. Langer , M. J. Mitchell , Nat. Rev. Drug Discovery 2019, 18, 175. 30622344 10.1038/s41573-018-0006-z PMC6410566

[31] X. Kong , Y. Qi , X. Wang , R. Jiang , J. Wang , Y. Fang , J. Gao , K. Chu Hwang , Prog. Mater. Sci. 2023, 134, 101070.

[32] M. A. Raheem , M. A. Rahim , I. Gul , X. Zhong , C. Xiao , H. Zhang , J. Wei , Q. He , M. Hassan , C. Y. Zhang , D. Yu , V. Pandey , K. Du , R. Wang , S. Han , Y. Han , P. Qin , OpenNano 2023, 12, 100152.

[33] M. Chountoulesi , D. R. Perinelli , A. Forys , V. Chrysostomou , A. Kaminari , G. Bonacucina , B. Trzebicka , S. Pispas , C. Demetzos , Int. J. Pharm. 2023, 630, 122440. 36436746 10.1016/j.ijpharm.2022.122440

