# Supplementary material for: Hybrid Poly(β‐amino ester) Triblock Copolymers Utilizing a RAFT Polymerization Grafting‐From Methodology
Source: Macromol Chem Phys. 2023 Nov 7;224(24):2300262. doi: 10.1002/macp.202300262 (PMC10941699; doi:10.1002/macp.202300262)
Supplement: Supplementary file 1 — Supporting Information [file MACP-224-2300262-s001.pdf]

Macromolecular Chemistry and Physics

Supporting Information

for *Macromol. Chem. Phys.*, DOI 10.1002/macp.202300262

Hybrid Poly(β-amino ester) Triblock Copolymers Utilizing a RAFT Polymerization Grafting-From Methodology

*Karolina Kasza, Amr Elsherbeny, Cara Moloney, Kim R. Hardie, Miguel Cámara, Cameron Alexander and Pratik Gurnani**

Supplementary information

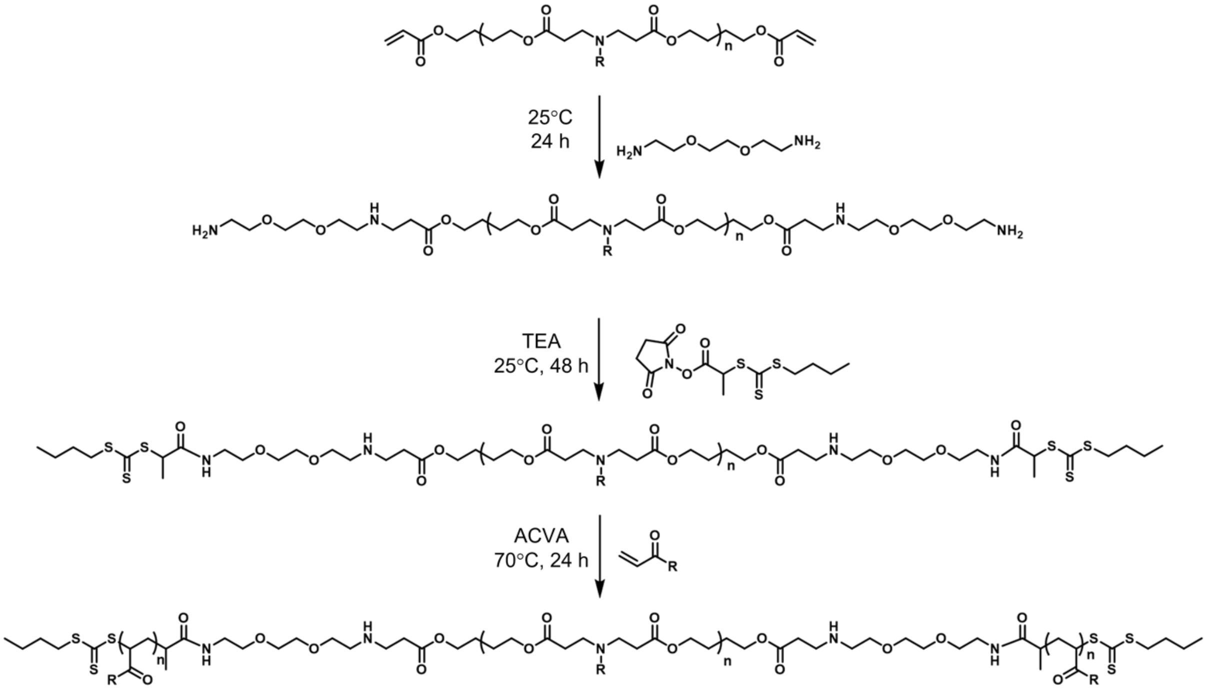

Figure S1: Synthetic scheme used to obtain PBAE-mCTA and its subsequent RAFT polymerisation.

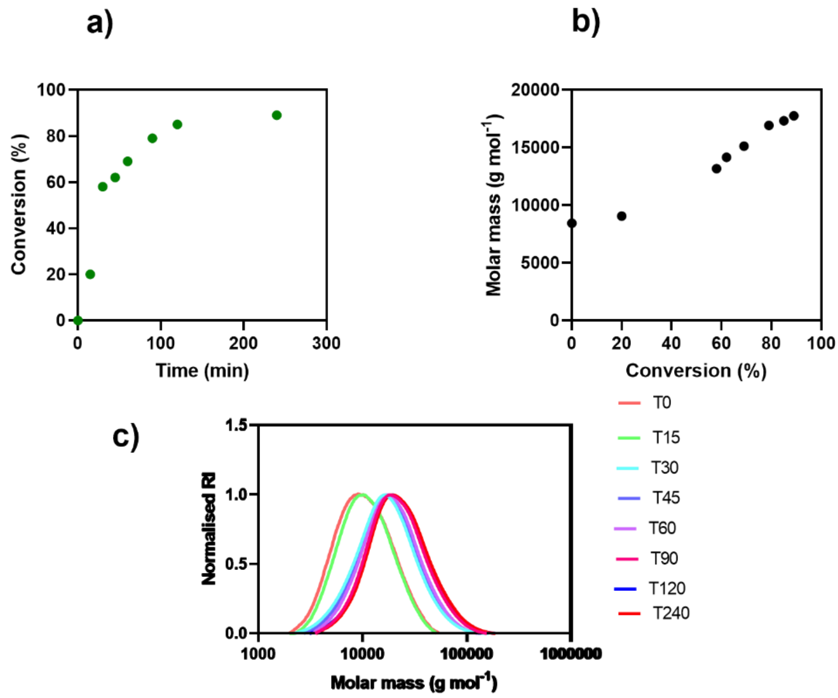

Figure S2: A) Evolution of monomer conversion of polymerization of NAM with BDD-3AP to obtain the NAM_{50}-(BDD-3AP)-NAM_{50} polymer. B) Liner evolution of molar mass (g mol^{-1}) with conversion. C) Evolution of SEC chromatograms over time.

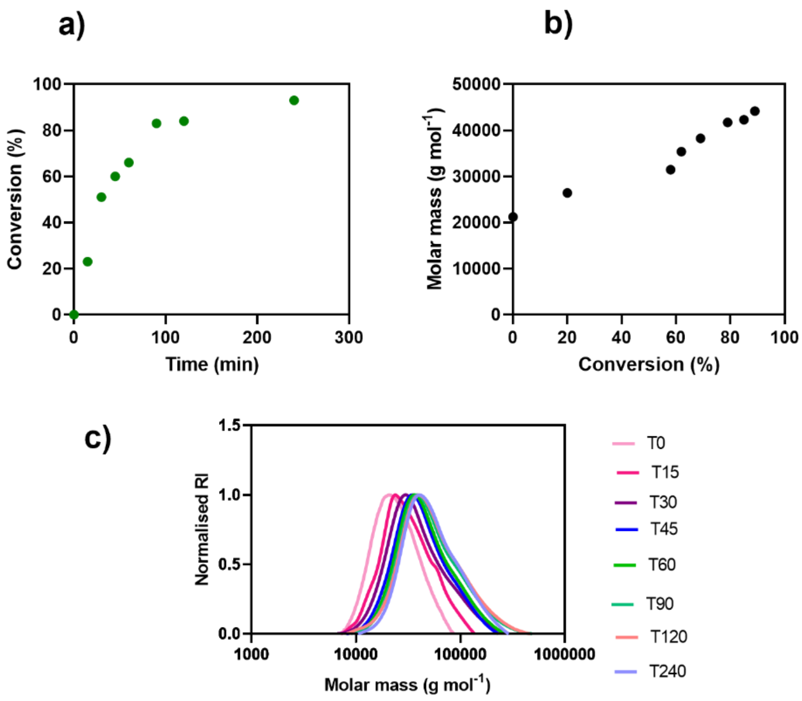

Figure S3: A) Evolution of monomer conversion of polymerization of NAM with HDD-PIP to obtain the NAM_{50}-(BDD-3AP)-NAM_{50} polymer. B) Liner evolution of molar mass (g mol^{-1}) with conversion. C) Evolution of SEC chromatograms over time.

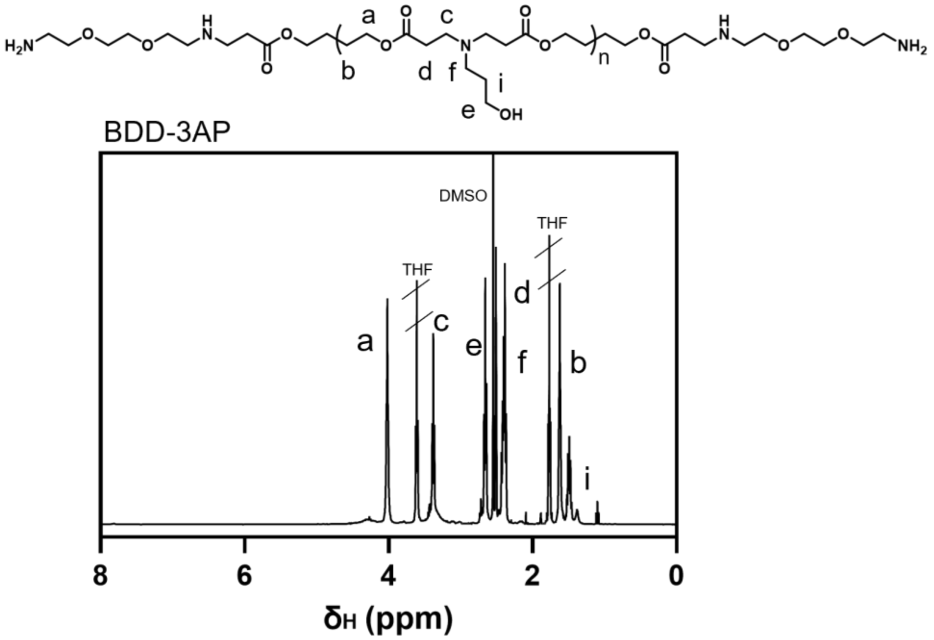

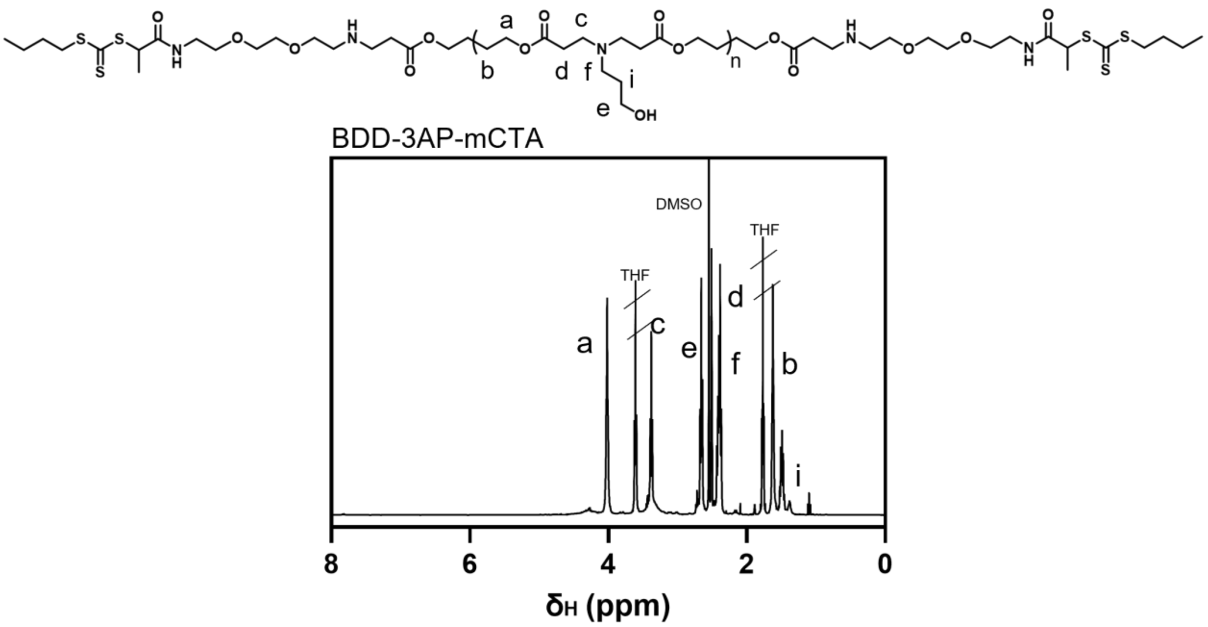

BDD-3AP-mCTA
DMSO
THF
a
b
c
d
e
f
i
n
8
6
4
2
0
δH (ppm)

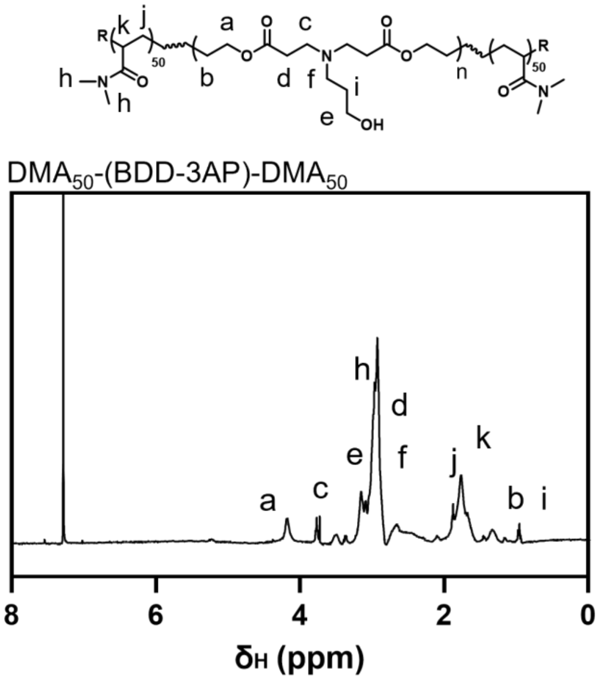

DMA50-(BDD-3AP)-DMA50
a
b
c
d
e
f
h
i
j
k
n
50
R
8
6
4
2
0
δH (ppm)

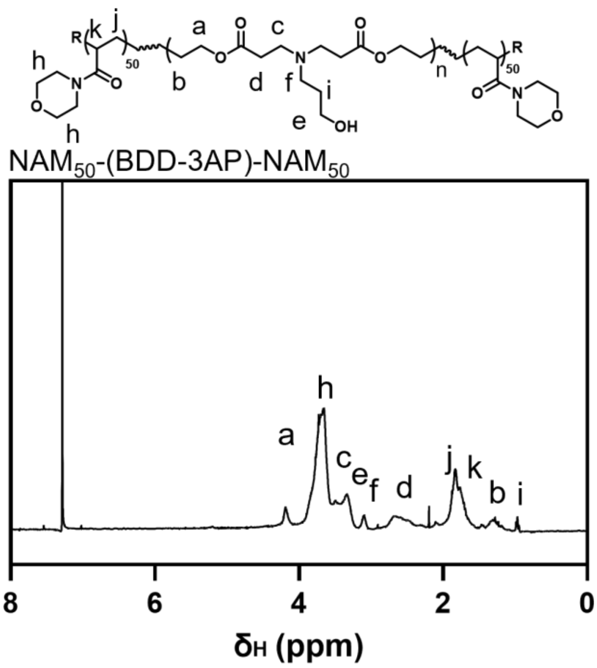
NAM50-(BDD-3AP)-NAM50
a
h
c
e
f
d
j
k
b
i
8
6
4
2
0
δH (ppm)

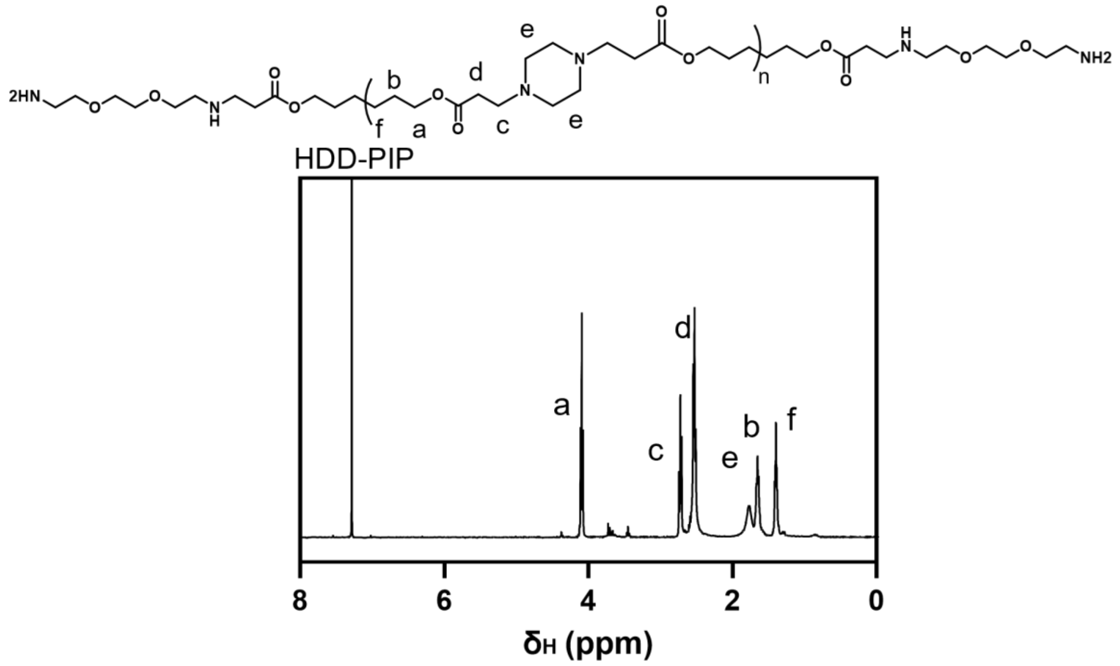
HDD-PIP
a
c
d
e
b
f
8
6
4
2
0
δH (ppm)

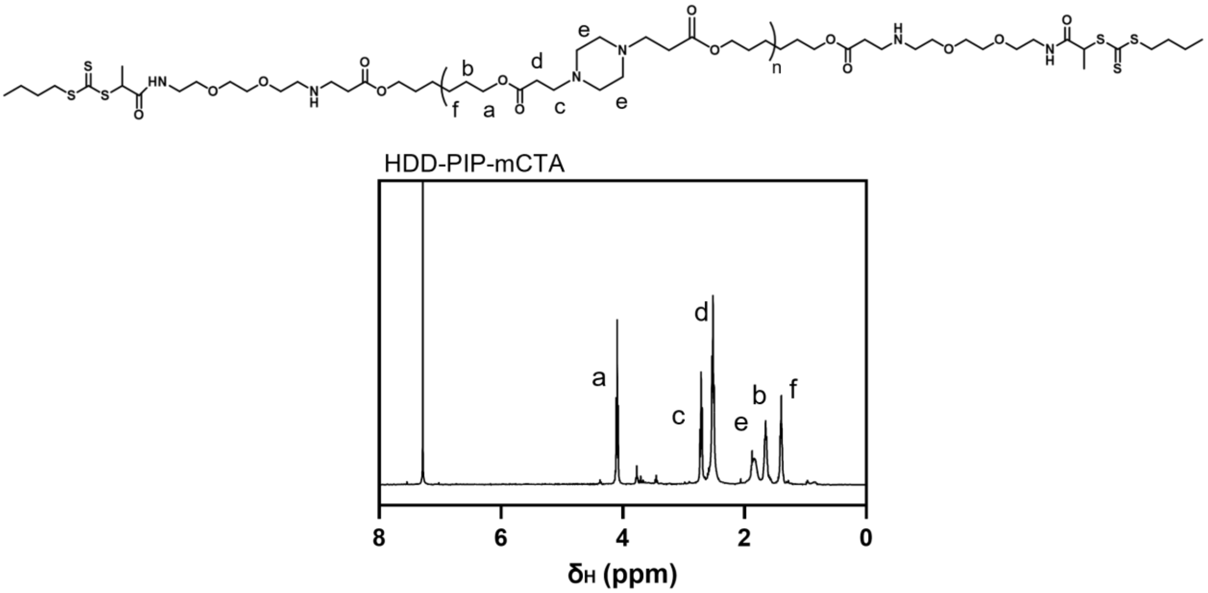
HDD-PIP-mCTA
a
b
c
d
e
f
n
δH (ppm)
8
6
4
2
0

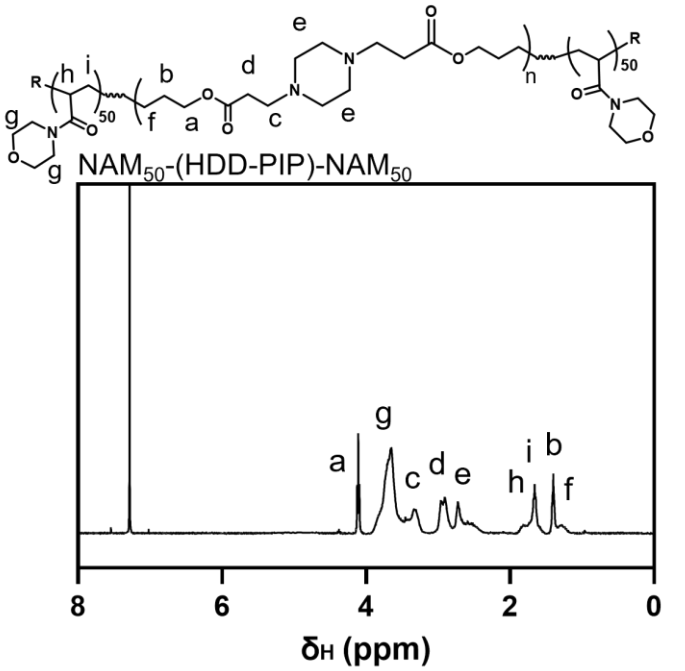
NAM50-(HDD-PIP)-NAM50
R
50
n
a
b
c
d
e
f
g
h
i
δH (ppm)
8
6
4
2
0

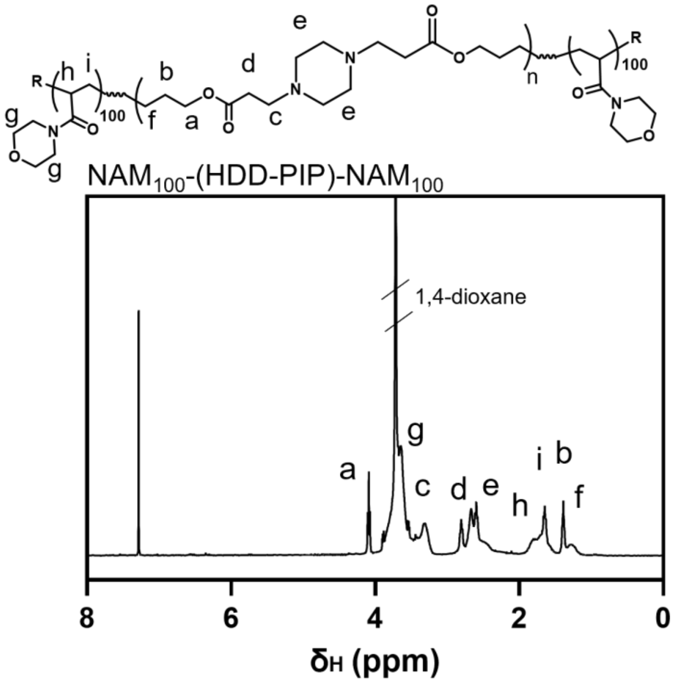
NAM100-(HDD-PIP)-NAM100
1,4-dioxane
a
g
c
d
e
h
i
b
f
8
6
4
2
0
δH (ppm)

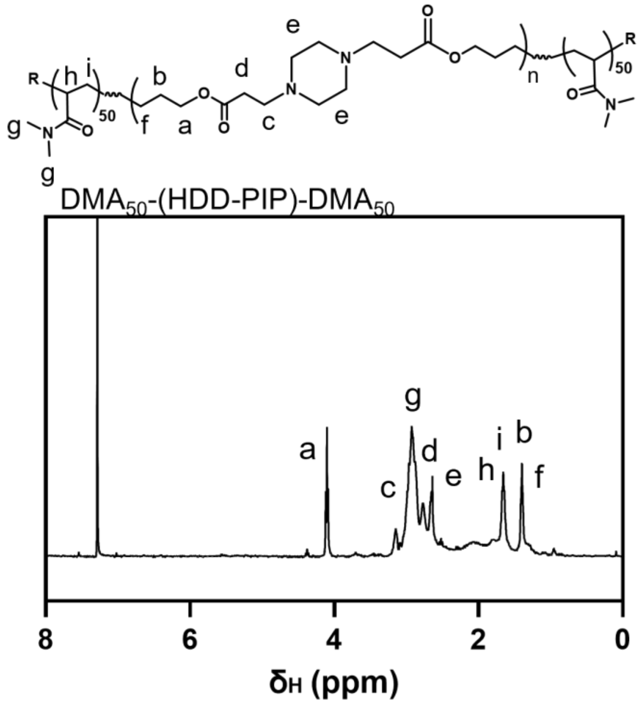
DMA50-(HDD-PIP)-DMA50
a
g
c
d
e
h
i
b
f
8
6
4
2
0
δH (ppm)

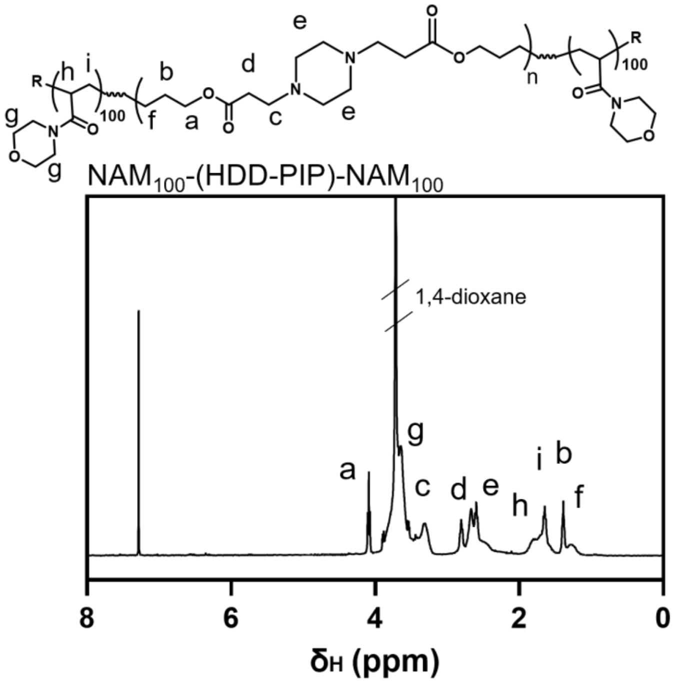

Figure S4: ^{1}H NMR spectra of polymers in DMSO-d6 or chloroform-d6 recorded at 25°C with a Bruker NMR400 MHz.

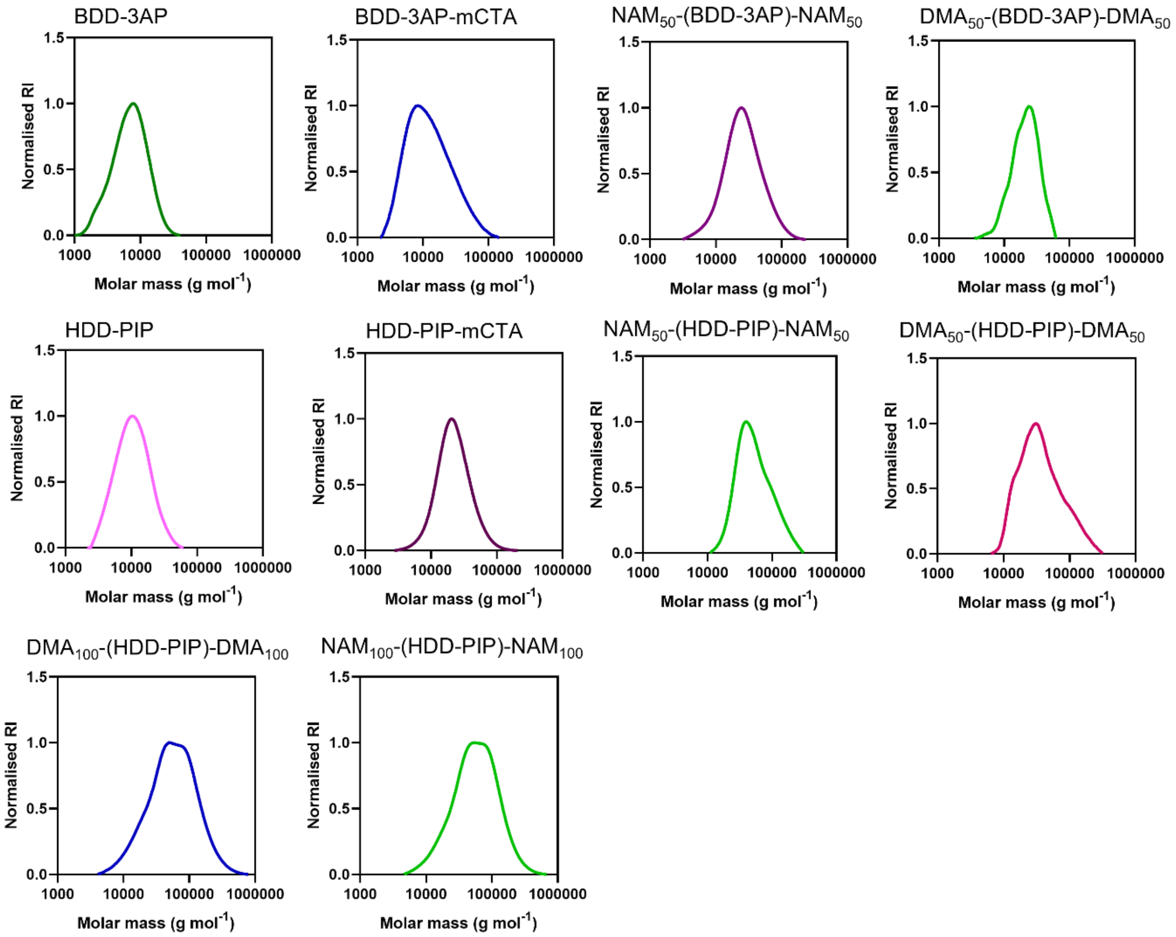

Figure S5: DMF-SEC chromatograms of prepared polymers.

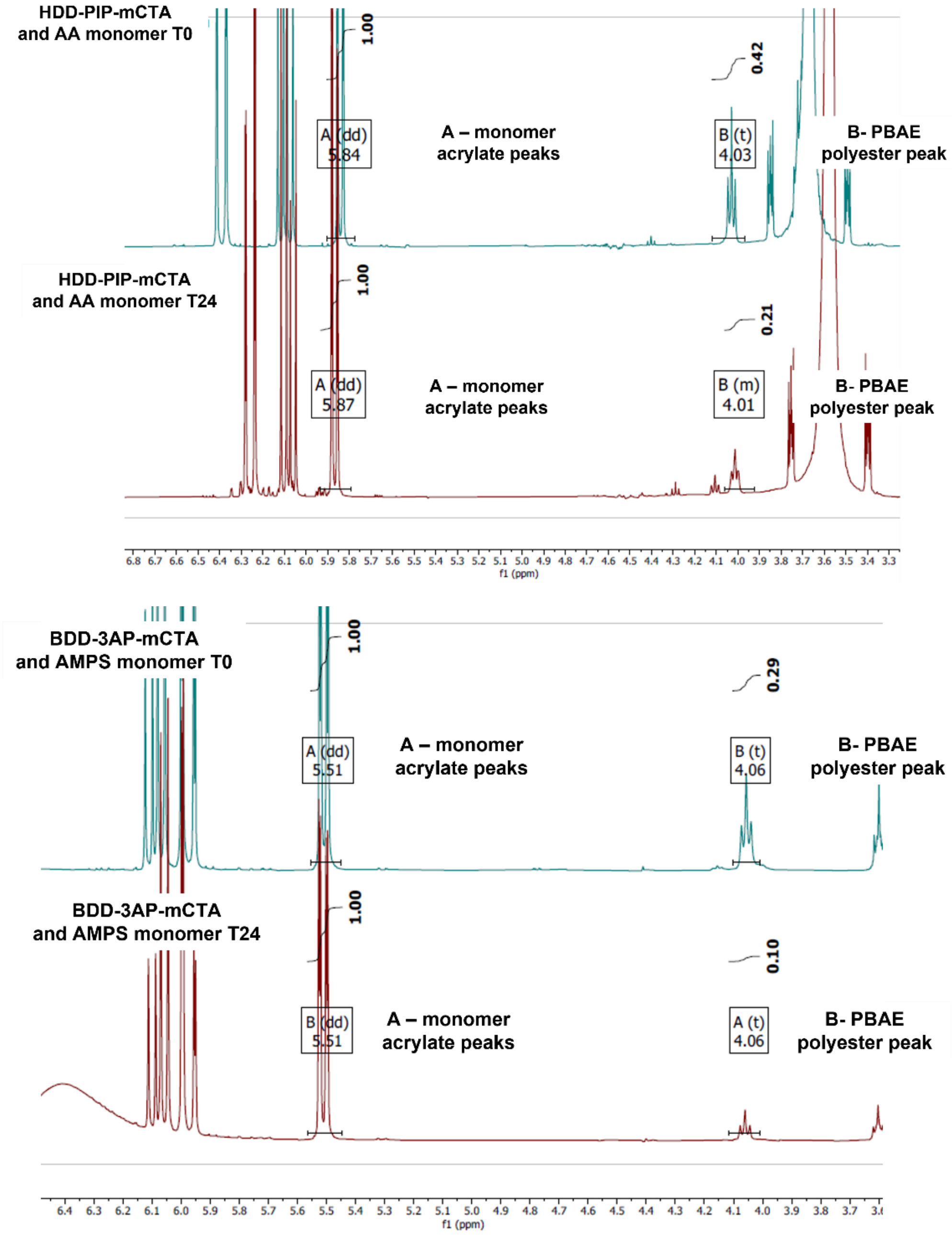

Figure S6: Degradation of PBAE by AA and AMPS monomers. Verified by integrating monomer peaks (around 5.5 ppm) as 1 and seeing the reduction in the integral corresponding to the PBAE polyester peak (around 4.01 ppm) following the 24 h RAFT polymerization.

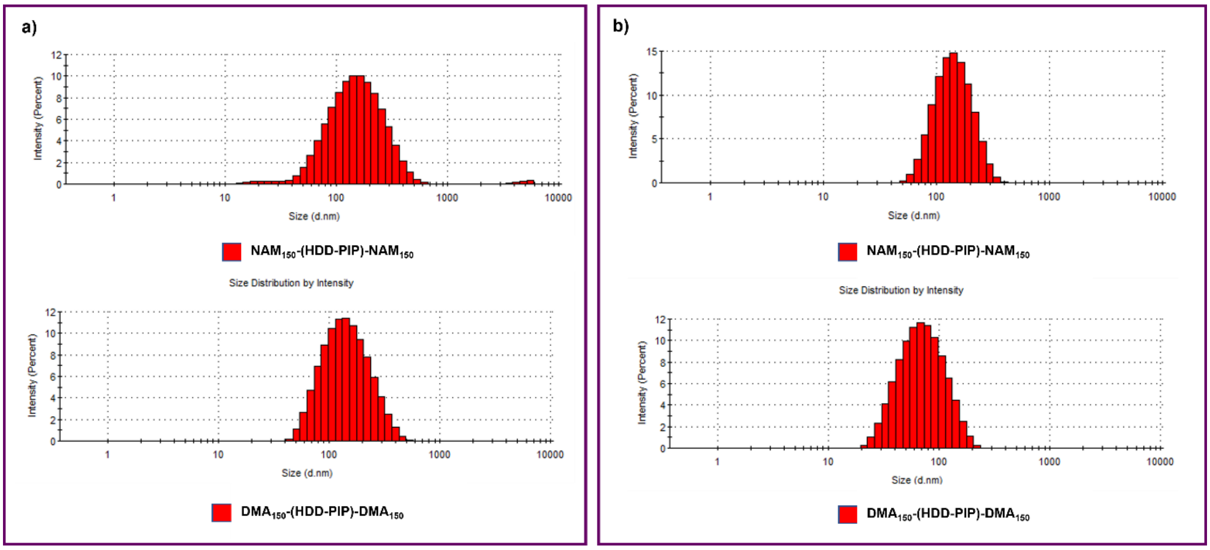

Figure S7: Change in particle size (nm) distribution by intensity (%) in water, at 1 mg mL^{-1}, across 48 h, where **a)** Particle size distribution at the 0 h timepoint; **b)** Particle size distribution at the 48 h timepoint.

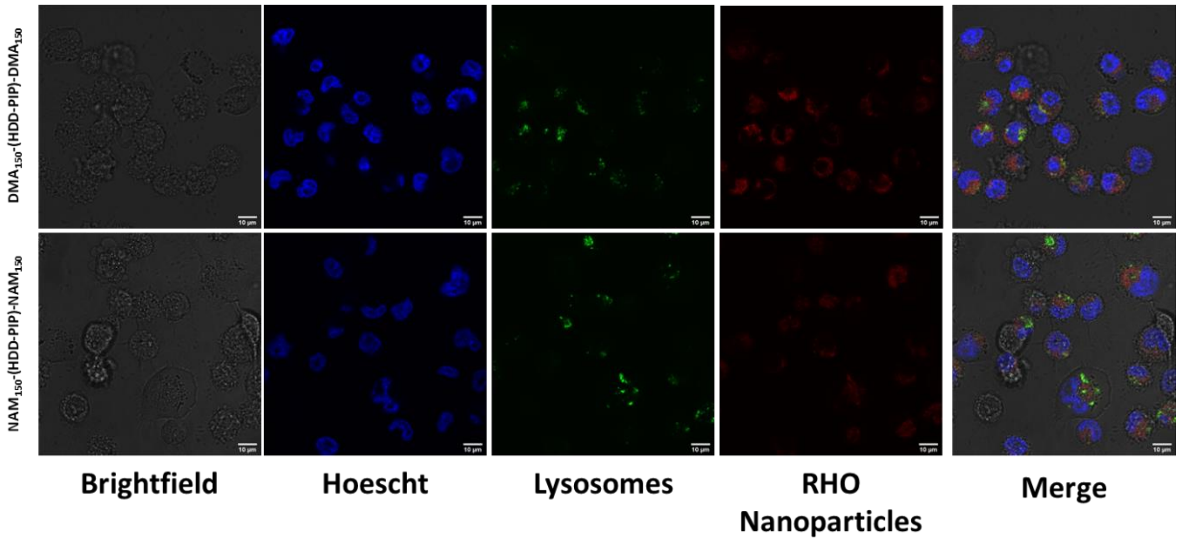

Figure S8: Original confocal microscopy images at 63x illustrating the cellular uptake of rhodamine labelled NAM150-(HDD-PIP)-NAM150 and DMA150-(HDD-PIP)-DMA150 nanoparticles in MDA-MB-31 breast cancer cell lines and their co-localisation within lysosomes (LumiTracker Lyso Green) before enhancing the contrast by saturating the pixels by 0.1% using the 'enhance contrast' feature in ImageJ. Scale bar = 10µm

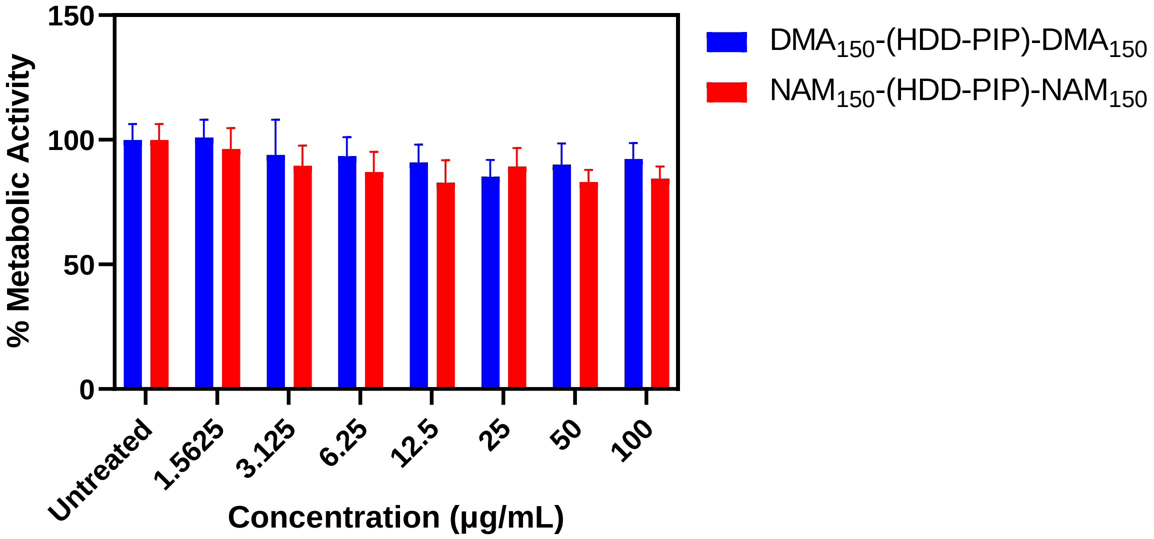

Figure S9: % Metabolic activity of blank NAM_{150}-(HDD-PIP)-NAM_{150} and DMA_{150}-(HDD-PIP)-DMA_{150} nanoparticles in MDA-MB-31 breast cancer cell lines after treatment for 72 h (n=5)
